# Emodin regulates cell cycle of non-small lung cancer (NSCLC) cells through hyaluronan synthase 2 (HA2)-HA-CD44/receptor for hyaluronic acid-mediated motility (RHAMM) interaction-dependent signaling pathway

**DOI:** 10.1186/s12935-020-01711-z

**Published:** 2021-01-06

**Authors:** Mingzhu Li, Shengbo Jin, Yang Cao, Jian Xu, Shendong Zhu, Zheng Li

**Affiliations:** 1 Department of Integrated Traditional Chinese and Western Medicine Medical Oncology, Cancer Hospital of China Medical University, Liaoning Cancer Hospital & Institute, Liaoning, China; 2 Traditional Therapy Center, Liaoning TCM Hospital, Liaoning, China; 3 Department of Gynecology, Cancer Hospital of China Medical University, Liaoning Cancer Hospital & Institute, Liaoning, China; 4 Department of Colorectal Surgery, Cancer Hospital of China Medical University, Liaoning Cancer Hospital & Institute, Liaoning, China; 5 Cancer Hospital of China Medical University, Liaoning Cancer Hospital & Institute, Liaoning, China

**Keywords:** NSCLC, HAS, CD44, RHAMM, Cell cycle

## Abstract

**Background:**

Non-small cell lung cancers (NSCLC) account for most cases of lung cancer. More effort is needed to research new drug and combination therapies for this disease. An anthraquinone derivative, emodin shows anticancer potency. We hypothesis that emodin suppresses lung cancer cells through hyaluronan (HA) synthase 2-HA-CD44/receptor for hyaluronic acid-mediated motility (RHAMM) interaction-dependent signaling pathway mediated cell cycle regulation.

**Methods:**

We tested the effect of emodin on viability, apoptosis, and HA secretion of 5 NSCLC cell lines. We used NSCLC cells A549 for two rounds of knockdown study: (1) knocking down either the synthases (HAS2 and HAS3) or the receptors (CD44 and RHAMM); (2) knocking down either HAS2 or HAS3. Then determined the effect of emodin on viability, HA secretion, cell cycle, and expression of cyclin proteins.

**Results:**

Emodin suppressed viability and HA secretion of all 5 NSCLC cell lines except for HA secretion of H460. Emodin had a slight apoptosis induction effect on all cell lines and was not different among cell lines. The knockdown of either the synthases or the receptors blocked emodin effects on viability while the knockdown of HAS2 block emodin effects but not HAS3. Emodin increased cells in the G1/G0 phase, and decreased cells in the S and G2/M phase by down-regulating cyclin A and B and up-regulating cyclin C, D, and E. HAS2 knockdown blocked the effects of emodin on the cell cycle.

**Conclusions:**

This study demonstrated that emodin regulates the cell cycle of NSCLC cells through the HAS2-HA-CD44/RHAMM interaction-dependent signaling pathway.

## Background

Lung cancer results in most cancer death among males and the second most cancer death among females in 2020 in the world [1]. Lung cancer rates are reducing year by year in most of the developed countries, such as the United States, United Kingdom, and Australia, but are elevating in low- and middle-income countries where smoking occurred later [1]. Non-small cell lung cancers account for about 85% of lung cancers, whereas small cell lung cancers only occupy approximately 15% of lung cancers [2]. Over the past two decades, a great improvement has been achieved in the clinical therapy of non-small cell lung cancer (NSCLC) [3], but, so far, the low rates of cure and survival for NSCLC patients urge more effort to research new drug and combination therapies for this disease.

Recently, many studies were developing naturally occurring compounds for clinical use [4–8]. An anthraquinone derivative, emodin (1,3,8-trihydroxy‐6‐methylanthraquinone), which is identified in Cassia obtusifolia [9], Aloe vera [10], Polygonum multiflorum [11], Rheum palmatum [12], and Polygonum cuspidatum [13], was thought to have multiple pharmacological effects. Emodin has been proved to have anti-cancer and anti-inflammatory properties [14, 15]. A study in breast cancer cell lines showed that emodin can inhibit MCF-7 growth and induce its apoptosis. In addition, liver cancer cells were also suppressed by emodin [16]. Emodin is included in some clinical traditional medicine prescriptions used for lung cancer in some Chinese hospitals. Therefore, we suggested that emodin might have inhibition toward lung cancer cells.

Hyaluronan (HA) is a molecule in the cancer micro-environment that is associated with malignancy. Transmembrane HA synthases 1–3 (HAS1, HAS2, or HAS3) is responsible for the synthesis of HA in mammalian cells [17]. After processed by hyaluronidases, mechanical forces, HA becomes a signaling molecule that can regulate inflammatory and tumorigenic [18]. HA interacts with cells through several cell surface receptors, the most critical of which is CD44 and the receptor for hyaluronic acid-mediated motility (RHAMM). Binding of HA to CD44/RHAMM on cells regulates cell proliferation by affecting a variety of downstream signaling pathways [19, 20]. Studies have revealed that HA is overexpressed in lung carcinoma over normal lung tissue [21]. Clinical data also suggested HA expression is associated with a higher frequency of recurrence [22]. CD44 and RHAMM are also overexpressed in lung cancer [23]and have been proved to correlate with worse cancer outcomes [24]. HA-CD44/RHAMM signal pathway has been reported to affect lung cancer proliferation [25].

Our preliminary experiments found that the HA expression of non-small lung cancer cells was affected by emodin, thus we hypothesis that emodin affects non-small lung cancer cells through HA CD44/RHAMM signaling pathway. In this study, we demonstrated the hypothesis and then knocked down critical targets of the HA CD44/RHAMM signaling pathway to explore the exact target of emodin. As we suggested the effect of emodin is mediating by HA cell cycle regulation, we analyzed the cell cycle and tested the cyclin proteins. Our study aims to deepen the understanding of the role of HA CD44/RHAMM signaling pathway in non-small lung cancer cells and develop emodin as a novel drug for the treatment of lung cancer.

## Methods

### Cell lines and cell culture

A549 (ATCC^®^ CCL-185™), H520 (ATCC^®^ HTB-182™), H1975 (ATCC^®^ CRL-5908™), H1299 (ATCC® CRL-5803™), H460 (ATCC^®^ HTB-177) were obtained from ATCC (Washington, USA). A549was cultured in Dulbecco’s Modified Eagle Media (DMEM) with 10% Fetal Bovine Serum (FBS) (ATCC^®^ 30-2020™). H520, H1975, H1299, and H460were cultured in RPMI-1640 medium (ATCC^®^ 30-2001™) with 10% FBS. Cell lines were cultured in a cell culture incubator with a humidified atmosphere of 5% CO_2_ at 37 °C.

### MTT assay

Cell viability was determined using The cells were plated in 96-well plates at 4 × 10^3^/well. Cells were cultured in the FBS-free medium for 24 h for starvation. After exposure to emodin for 24 h, methylthiazoletetrazolium (MTT, Abcam, Cambridge, UK) was incubated with the cells (40 µL /well) for 4 h. A microplate reader was used to read the plate at 490 nm.

### ELISA assay

The concentration of HA in the culture medium was determined by using Hyaluronan Quantikine ELISA Kit (Minneapolis, MN, US). The apoptosis of the cells was also determined by ELISA assay. The cells were plated in 96-well plates (3–5 × 103/well) for 12 h and were treated as described before. The apoptosis assays were then performed using Cell Death Detection ELISA plus (Roche, Indianapolis, IN, USA), which monitors DNA fragmentation. The absorbance was measured at 490 nm using the Multiskan™ FC Microplate Photometer. We prepared a positive control for each cell line by inducing cell death through incubating cells at 55 ◦C for 20 min.

### Cell transfection

Gene knockdown in A549 was achieved by siRNA transfection. The transfection method was described previously [26]. Briefly, cells were transfected with siRNA or negative siRNA using Lipofectamine^®^ 2000. The concentration and time of transfection for the siRNA was 50 nM for 72 h. The expression of target proteins in cells was validated by western blotting assay. The Lipofectamine^®^ 2000 kite, HAS2 siRNAs (AM16708), HAS3 siRNA (AM16708), CD44 siRNA (AM16708), and negative siRNA (AM4611) were obtained from ThermoFisher Scientific (Waltham, MA, USA). RHAMM siRNA (sc-40181) were obtained from Santa Cruz Biotechnology (Dallas, TX, US).

### Cell cycle assay

The cell cycle was determined using flow cytometry with propidium iodide (PI) staining, which was described previously [27]. 4 × 10^6^ suspended cells were fixed with pre-cold 70% ethanol for 2 min at 4 °C. The cells were then mixed with 0.02 mg/mL DNAse-free RNAse A with 0.2 mg/mL 0.1% Triton X-100 in cold PBS at 37 °C for 30 min. Cells were detected with BD FACSCalibur (Becton Dickinson. San Jose, CA, USA).

### Western blotting

Protein expression was determined by western blotting, which was described previously [28]. Cells were lysed with RIPA buffer with protease inhibitor (Sigma-Aldrich, USA). Total protein concentrations were tested by BCA. SDS gel electrophoresis was conducted to separate the proteins with a loading amount of 30 µg/well). Then the proteins were electrically transferred onto polyvinylidene difluoride membranes (0.2-µm). The membranes were incubated with a blocking buffer (5% skimmed milk in TBS). Membranes were then incubated with primary (1:1000 dilution of BSA, 4 °C, overnight) and secondary antibodies (1:3000 dilution of BSA, room temperature, 2 h). ECL was used to visualize the proteins. The proteins were quantified by Image Studio™ Lite (Lincoln, NE, US). Anti-cyclin A antibody (sc-271,682), Anti-Cyclin B antibody (ab72), Anti-Cyclin C antibody (ab85927), Anti-Cyclin D1 antibody (ab40754), Anti-Cyclin E1 antibody (ab133266), and all the secondary antibodies were purchased from Abcam (Cambridge, UK).

### Statistics and plotting

T-test or one-way ANOVA and Dunnett’s post hoc tests were used to analyze the significance of the difference. p-value greater than 0.05 was considered a significant difference.

## Results

### Emodin suppressed the viability of lung cancer cells with different potency

**Fig. 1:**
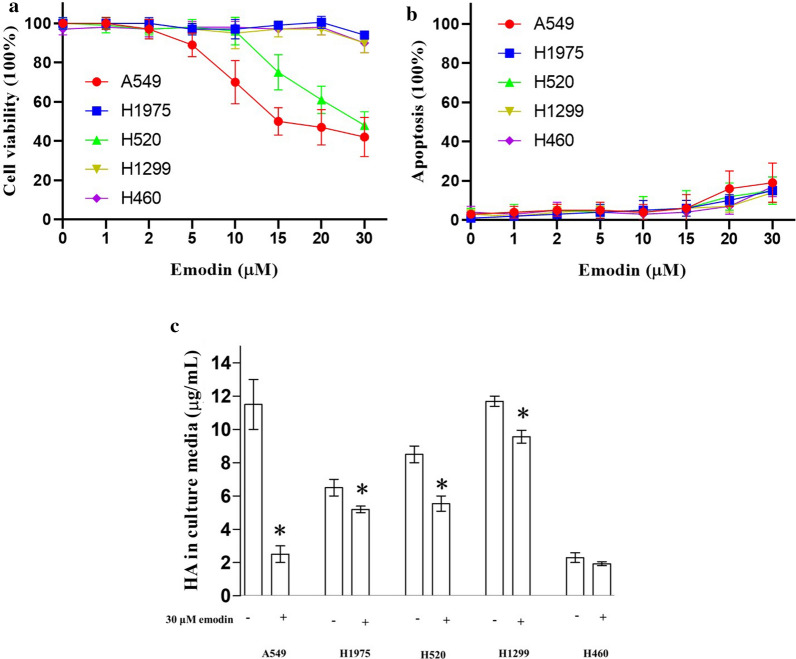
Effect of emodin on the cell viability and HA secretion of lung cancer cells. Lung cancer cell lines were exposed to 0–30 µM emodin for 24 h before the MTT and ELISA assay. **a** Suppression of emodin on cell viability of lung cancer cell lines. A549 was the most sensitive cell line with an effective concentration of 5 µM. Viability of A549 was dose-dependently inhibited by emodin at 5–30 mM. H520 was the second most sensitive cell line with an effective concentration of 15 µM. Viability of H520 was dose-dependently inhibited by emodin at 15–30 mM. The other three cell lines, including H1975, H1299, and H460, showed a significant decrease in cell viability only when the concentration of emodin reached 30 µM. **b** The effect of emodin on apoptosis. Emodin at 20–30 mM had a slight apoptosis induction effect (< 20%) on all cell lines. The apoptosis induction of emodin was not different among cell lines. **c** The effect of emodin on HA secretion of lung cancer cells. Emodin at 30 µM suppressed HA secretion in all lung cancer cell lines tested except for H460, inferring that emodin might regulate HA generation. A549 was the most sensitive cell line with a suppression rate of over 75%. “*” marks the significant difference (p < 0.05) compare with the control (0 µM or “−”)

Emodin at 20–80 µM was reported to suppress the viability of liver cancer cells [16]. Our preliminary data showed that emodin at 10–100 µM suppressed the viability of lung cancer cells with different potency (data not shown). In this study, we used a lower concentration range (1–30 mM) aiming to distinguish the potency of different cell lines. We used MTT assay to determine the effect of emodin at 1–30 µM on cell viability of five lung cancer cell lines A549, H520, H1975, H1299, and H460. MTT assay is commonly used for cancer pharmacological studies [29]. Results show that A549 was the most sensitive cell line with an effective concentration of 5 µM. Viability of A549 was dose-dependently inhibited by emodin at 5–30 mM. H520 was the second most sensitive cell line with an effective concentration of 15 µM. Viability of H520 was dose-dependently inhibited by emodin at 15–30 mM. The other three cell lines, including H1975, H1299, and H460, showed a significant decrease in cell viability only when the concentration of emodin reached 30 µM (Fig. 1a). Besides, we also determined the effect of emodin on apoptosis. Emodin at 20–30 mM had a slight apoptosis induction effect (< 20%) on all cell lines. The apoptosis induction of emodin was not different among cell lines (Fig. 1b). Therefore, we suggested the difference in the viability of cell lines resulted from the effect of emodin on cell proliferation. Emodin at 30 µM showed the most difference in the effect on viability among lung cancer cell lines, thus it was used in the subsequent study.

### Emodin suppressed the secretion of HA in lung cancer cell lines

To demonstrate our hypothesis, we tested the effect of emodin on the ability of lung cancer cells to produce HA. The concentrations of HA in the culture media, which we measured by ELISA, indicated the expression of HA. Results showed that Emodin at 30 µM suppressed HA secretion in all lung cancer cell lines tested except for H460, inferring that emodin might regulate HA generation. A549 was the most sensitive cell line with a suppression rate of over 75%. (Fig. 1b) As A549 was the most sensitive to emodin both in cell viability and HA secretion, it was used in the subsequent knockdown study.

### The sensitivity of viability suppression was associated with the sensitivity of suppression of the HA secretion

**Fig. 2:**
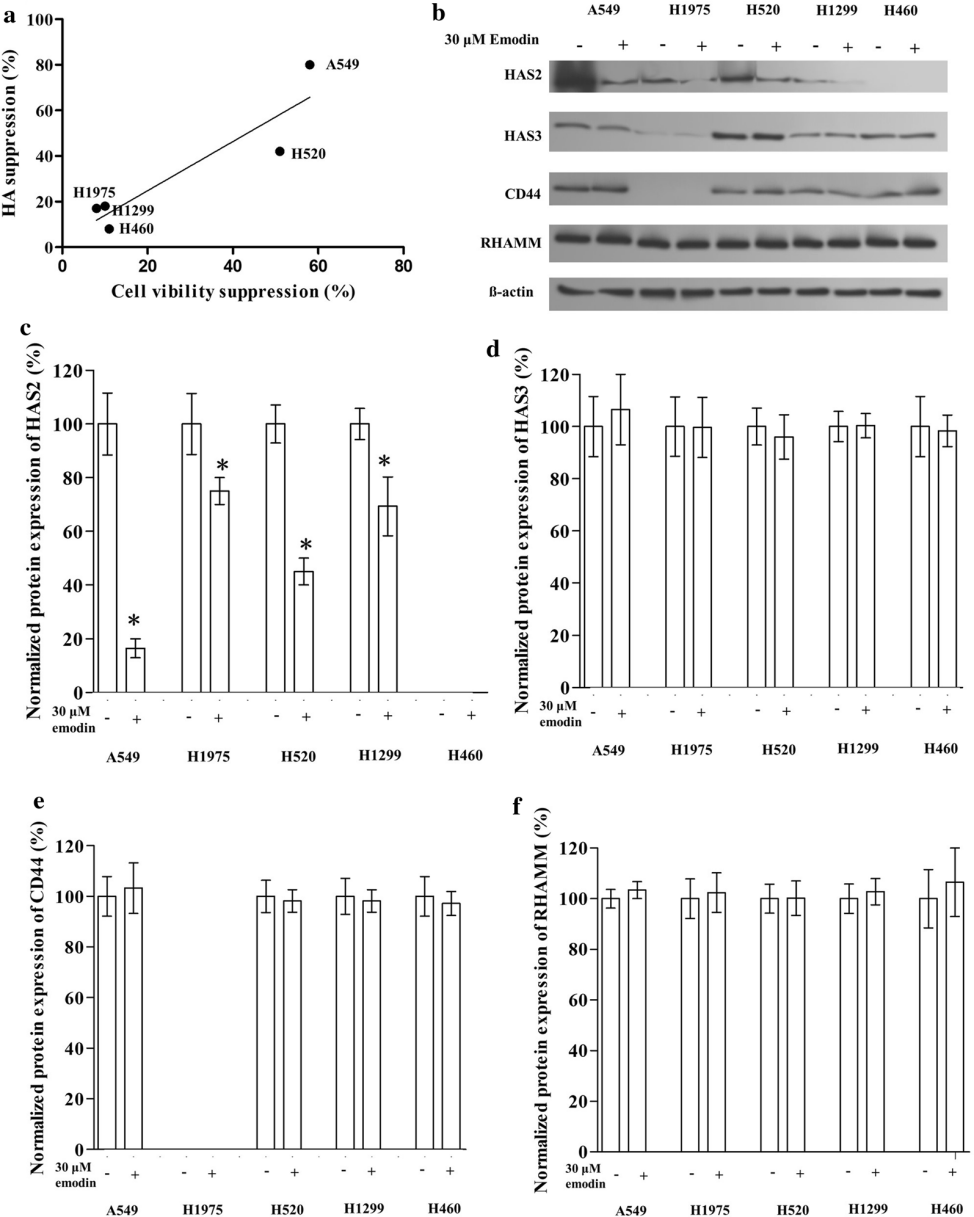
Effect of emodin on the HA-CD44/RHAMM signaling pathway in lung cancer cell lines. Cells were exposed to 30 µM emodin for 24 h before the western blotting assay. **a** The suppression rate of emodin on cell viability was plotted vs. suppression rate of emodin on HA secretion. **d** Representative image of western blotting. **c–f** The effect of emodin on the protein expression of HAS2, HAS3, CD44, and RHAMM in lung cancer cell lines (Bar chart for western blotting). “*” marks the significant difference (p < 0.05) compare with the control (0 µM or “−”)

Among the five lung cancer cell lines tested, we found a rough correlation between the suppression of emodin on cell viability and that on HA secretion (Fig. 2a). A549 and H520 had a relatively higher suppression rate of emodin on cell viability, which was also more sensitive in terms of emodin secretion suppression. This suggested a potential association between emodin suppression on HA generation and that on cell viability.

### Emodin suppressed HAS2 but not HAS3, CD44, and RHAMM in lung cancer cells

We determined the expression level of HAS2, HAS3, CD44, and RHAMM in lung cancer cell lines except for CD44 in H1975 and HAS2 in H460 which might be too low to be detected in our experiment. Results showed that emodin decreased the expression of HAS2 in lung cancer cells but had no significant effect on HAS3, CD44, and RHAMM. Notably, A549 had the highest expression level of HAS2 and also had the largest decrease in HAS2 level after exposed to emodin (Fig. 2b–f).

### Effect of knockdown of HAS2, HAS3, CD44, and RHAMM on A549

**Fig. 3:**
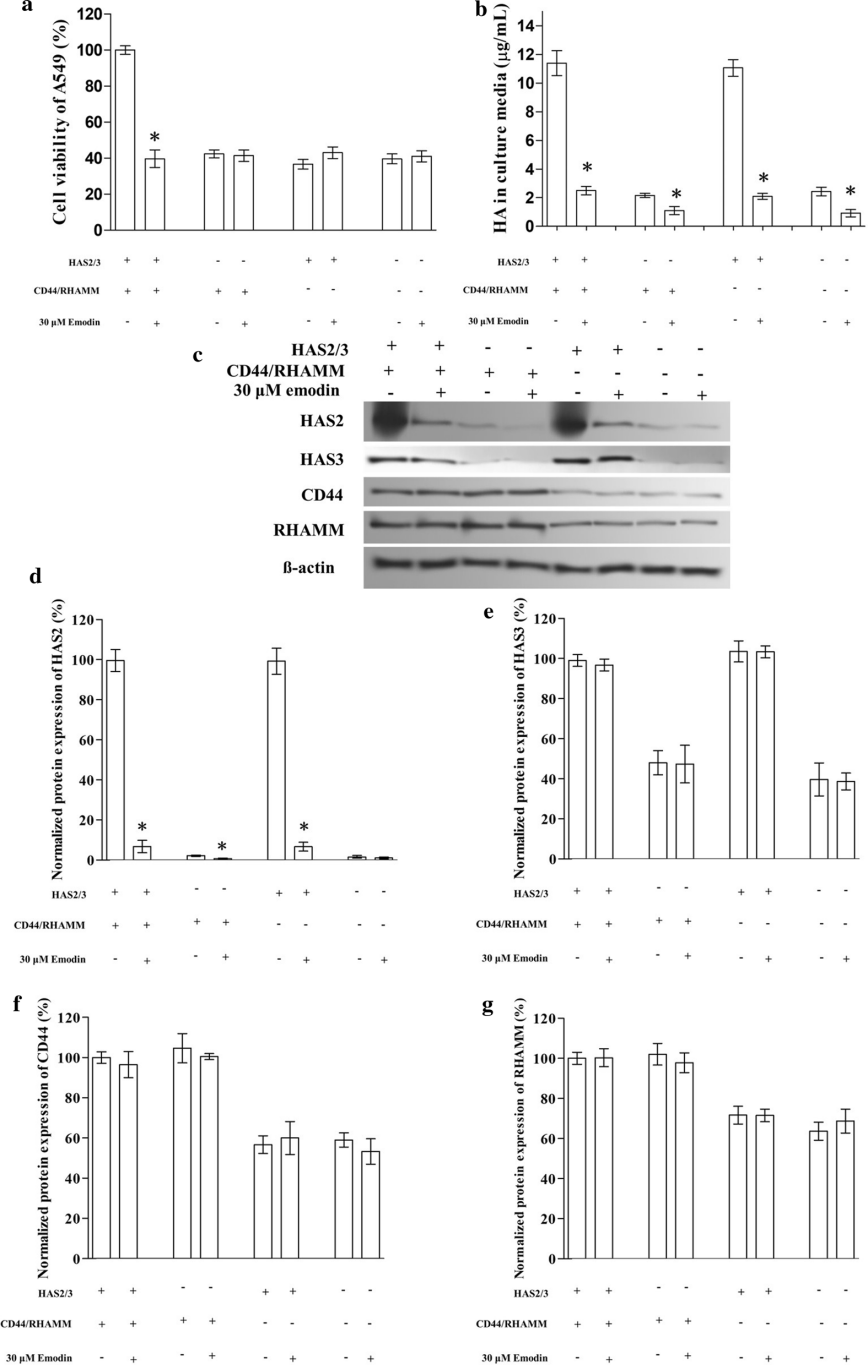
The effect of emodin on the HA-CD44/RHAMM signaling pathway knocked down A549 cells. The expression of HAS2 and HAS3 (HAS2/3) or CD44 and RHAMM (CD44/RHAMM) were knocked down in A549 cells. **a** The effect of emodin on viability. The transfected cells were exposed to 30 µM emodin for 24 h before the MTT assay. **b** The effect of emodin on HA secretion of transfected A549 cells. **c** Representative image of western blotting. **d**–**g** The effect of emodin on the protein expression of HAS2, HAS3, CD44, and RHAMM in transfected A549 (Bar chart for western blotting). “*” marks the significant difference (p < 0.05) compare with the vehicle control (“−”)

To test the hypothesis, we tried to block the HA-CD44/RHAMM signaling pathway by knocking down HAS2, HAS3, CD44, and RHAMM in A549. Western blocking confirmed that the protein expressions of HAS2, HAS3, CD44, and RHAMM were reduced in A549. The knockdown resulted in a dramatic decrease in HA secretion and also inhibited the viability by about 60%. The exposure of emodin further decrease the HA secretion but failed to impact the viability of the cells (Fig. 3).

### Effect of knockdown of HAS2 and HAS3 on A549

To specify the exact target of emodin, we first knocked down the expression of both HAS2 and HAS3 in A549. The knockdown of HAS2 and HAS3 decreased the viability and HA secretion of A549 by about 60% and 80% respectively. Then we exposed the cells to emodin and found that knockdown of HAS2 and HAS3 resulted in the decrease in HA secretion and the resistance of A549 to emodin viability suppression (Fig. 3).

### Effect of knockdown of CD44 and RHAMM on A549

Next, we knocked down the expression of two downstream targets, CD44 and RHAMM, in A549. The knockdown of CD44 and RHAMM decreased the viability to a similar level as HAS2 and HAS3 knockdown cells while HA secretion was not affected by the knockdown of CD44 and RHAMM. After exposed to emodin, HA secretion decreased by more than 80% but the viability of CD44 and RHAMM knocked down A549 cells was not affected significantly. (Fig. 3). Hence, we suggested that emodin impact the expression of HAS2 or HAS3 but not CD44 or RHAMM.

### Emodin suppressed the viability of A549 by suppressing HAS2 expression but not HAS3

**Fig. 4:**
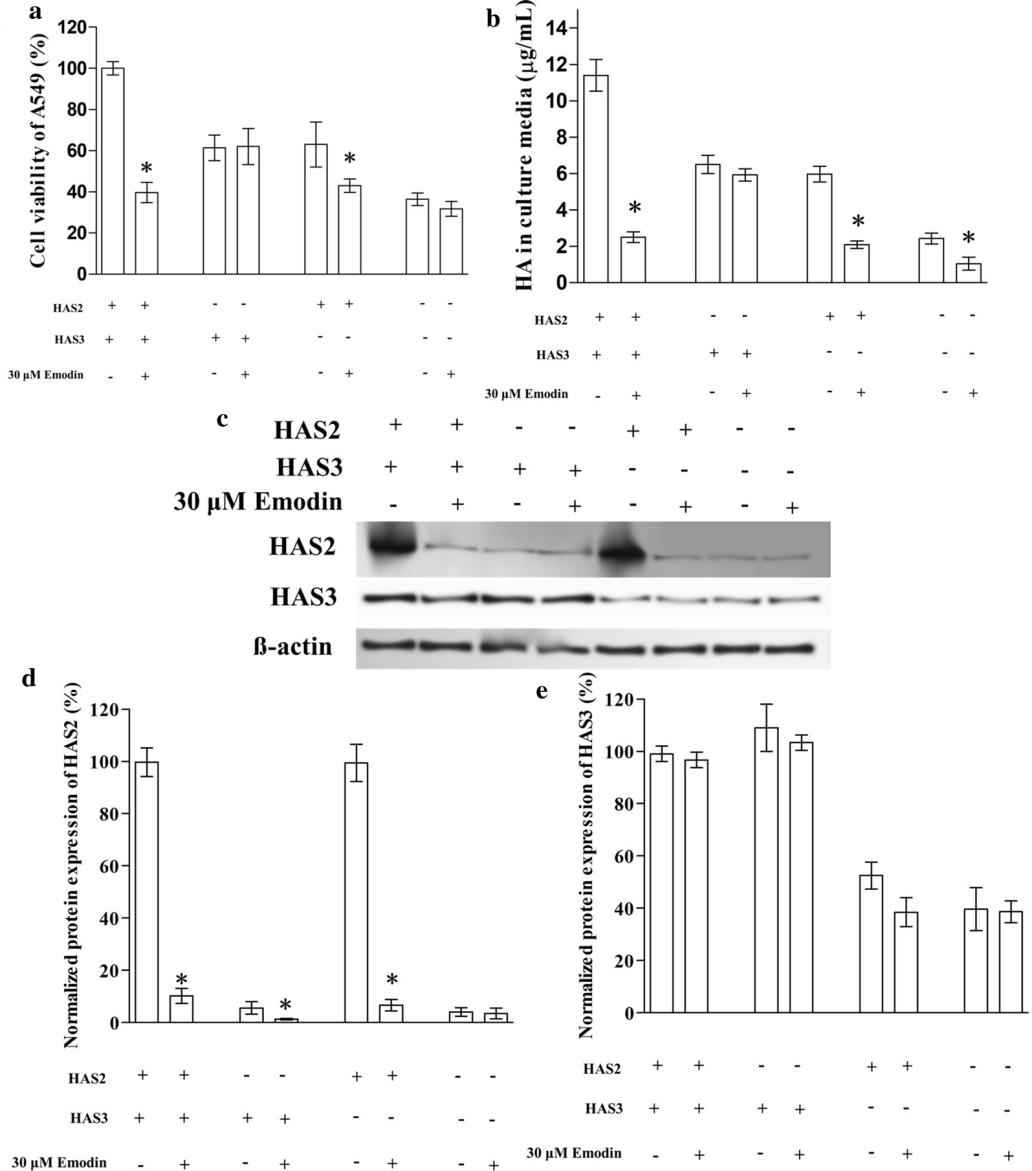
The effect of emodin on HAS2 or HAS3 knocked down A549 cells. The expression of HAS2 or HAS3 was knocked down in A549 cells. **a** The effect of emodin on the viability of knocked down A549 cells. The transfected cells were exposed to 30 µM emodin for 24 h before the MTT assay. **b** The effect of emodin on HA secretion of transfected A549 cells. **c** Representative image of western blotting. DE. The effect of emodin on the protein expression of HAS2 and HAS3, in transfected A549 (Bar chart for western blotting). “*” marks the significant difference (p < 0.05) compare with the vehicle control (“−”)

To further specify the exact target of emodin, we knocked down the expression of either HAS2 or HAS3 in A549 respectively. The western blotting confirmed that that knockdown was successful but the decrease of HAS2 (by about 90%) was more dramatic than the decrease of HAS3 (by about 50%) (Fig. 4c–e). The knockdown of HAS2 or HAS3 showed a similar effect: both the viability and HA secretion of A549 decreased by about 40%, but after the cells were exposed to emodin, the viability and HA secretion significantly decreased only in HAS2 knocked down cells but not in HAS3 knocked down cells (Fig. 4a, b).

### Emodin affect the cell cycle of A549 through HAS2

**Fig. 5:**
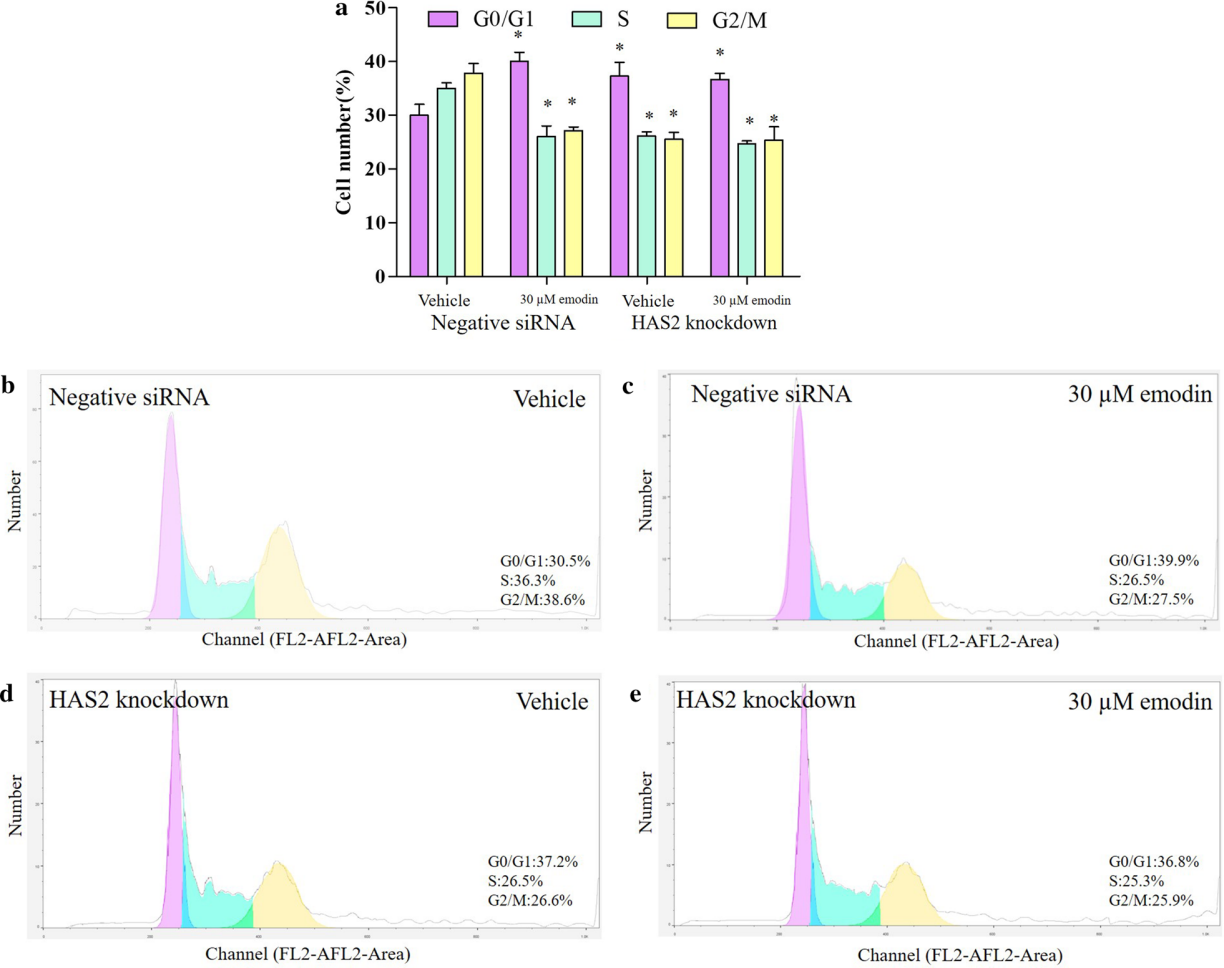
Effect of emodin on the cell cycle of A549. **a** The cell cycle was analyzed by flow cytometry. “*” indicates a significant difference compared with the vehicle of negative siRNA groups (p < 0.05). **b**–**e** Representative data of cell cycle

According to viability results, emodin at 30 µM suppressed the viability of A549, which, we suggested, was associated with the cell cycle regulation. We analyzed the cell cycle of A549 cells. In negative siRNA groups, emodin significantly increased cells in the G1/G0 phase and decreased cells in the S and G2/M phase. This result indicated that emodin arrested cell proliferation. HAS2 knockdown showed similar effects on the cell cycle, causing an increase in cells in the G1/G0 phase, and a decrease in cells in the S and G2/M phase. The knockdown of HAS2 also blocked the effect of emodin, suggesting that emodin affects the cell cycle of A549 through HAS2 (Fig. 5).

### Emodin regulated cyclin proteins through emodin in A549

**Fig. 6:**
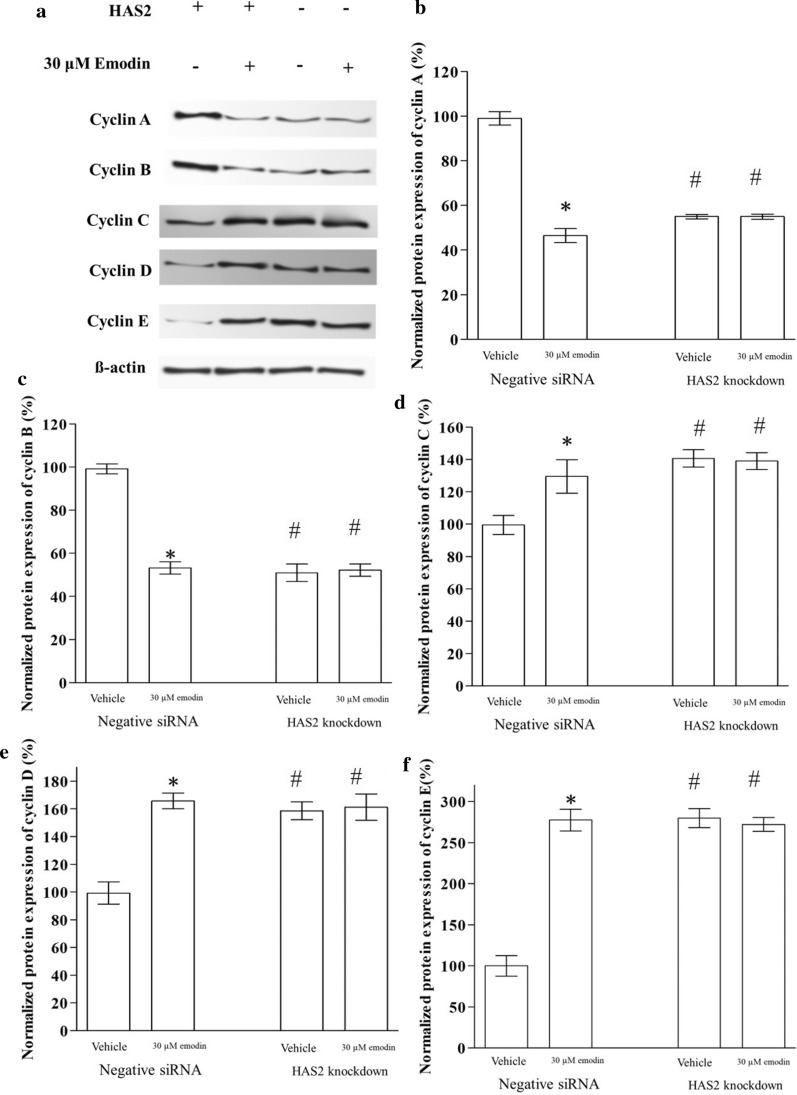
Effect of emodin on the expression of cyclin proteins in A549. **a** Images of western blotting results. **b**–**f** The effect of emodin on the protein expression of cyclin A, cyclin B, cyclin C, cyclin D, and cyclin E in A549 (Bar chart for western blotting). “*” marks the significant difference (p < 0.05) compare with the vehicle control. “#” indicates a significant difference (p < 0.05) compare with the vehicle’s negative siRNA control

To further explore the mechanism of emodin regulation on the cell cycle, we determined the expression of key regulatory proteins of the cell cycle by western blotting: cyclin A, cyclin B, cyclin C, cyclin D, and cyclin E. In negative siRNA control groups, emodin decreased the expression of cyclin A and cyclin B and increased cyclin C, cyclin D, and cyclin E. The HAS2 knockdown also emodin decreased the expression of cyclin A and cyclin B and increased cyclin C, cyclin D, and cyclin E. In the HAS2 knockdown groups, these cyclin proteins were not changed by emodin, proving that HAS2 mediated the effect of emodin on the cell cycle by regulating cyclin proteins (Fig. 6).

## Discussion

This study reveals the inhibition of emodin against the proliferation of non-small lung cancer cells. MTT assay, which is a common assay used in cancer cell viability studies [29], was used to screen five cell lines at different concentrations. The sensitive concentration range of emodin toward non-small lung cancer cells is similar to that of liver cancer cell line SMMC-7721, whose viability was suppressed by 20 µM emodin by about 30% [16]. Yet, by comparing the effect of emodin on five non-small lung cancer cell lines, we found that different cell lines we tested had different sensitivity toward emodin. This can account for the fact that the clinical treatment of emodin-containing traditional medicine only effective in some cases.

The non-small lung cancer cell lines used in this study are commonly used non-small-cell lung cancer cell lines derived from different sources. A549 cell line was developed from cancerous lung tissue in the explanted tumor of a 58-year-old caucasian male [30]. The H1975 cell line is used for the study of drug resistance based on some special mutations [31]. The H520 cell line was derived taken from a patient with squamous cell carcinoma of the lung. H520 cells express greatly reduced levels of p53 mRNA relative to normal lung tissue [32]. H1299 is a human non-small cell lung carcinoma cell line derived from the lymph node [33]. H1299 has a homozygous partial deletion of the TP53 gene [34]. H460 is a highly lymphomatous metastatic subline of a human non-small lung cancer cell line [35]. We believe using multiple cell lines can better demonstrate the effect of emodin is common among many Non-small-cell lung cancer cell lines. Besides, with multiple cell lines, we can select the most sensitive cell line for the subsequent study as it facilitates our observation. We did not know what caused the difference in the effect of emodin on HA secretion, but we found a rough correlation between the suppression of emodin on cell viability and that on HA secretion (Fig. 2a). A549 and H520 had a relatively higher suppression rate of emodin on cell viability, which was also more sensitive in terms of emodin secretion suppression. This suggested a potential association between emodin suppression on HA generation and that on cell viability. The effect of HA is largely through the activation of CD44/RHAMM [19, 20]. Thus, we hypothesis that emodin affects non-small lung cancer cells through the HA CD44/RHAMM pathway.

We designed a protein knockdown experiment to explore the target of emodin. We selected A549, in which we suggested HA CD44/RHAMM pathway might play the most role among cell lines we tested, for the knockdown experiments because it is the most sensitive cell line toward emodin in the MTT assay and HA detection. In the preliminary study, for some reason, A549 was not able to survive when we knocked down HAS1. A previous study studying HA CD44/RHAMM pathway in lung cancer cells only knocked down HAS2 and HAS3 as us [25], we suggested they might also come across the same issue. In the present study, there were two rounds of the knockdown experiment, in the first round, we knocked down either the synthases (HAS2 and HAS3) or the receptors (CD44 and RHAMM). Results showed that either of the knockdowns blocked the effects of emodin on A549 viability completely, suggesting that those targets were critical for the pharmacological effects of emodin. Either of the knockdowns also led to the decrease of cell viability indicating that the HA CD44/RHAMM pathway promotes cell viability, which is consistent with a previous study [25]. In the second round of the knockdown experiment, we knocked down either one of the synthases, HAS2 or HAS3. Results revealed that the effect of emodin was only associated with the expression of HAS2, indicating that emodin suppressed only HAS2 but not HAS3. Regarding the data of the other lung cancer cell lines we tested, in H1975, the expression of CD44 was low, indicating the absence of HA CD44/RHAMM pathway; while in H1299 and H460, the expression of HAS2 was low, resulting in the low sensitivity of the cell toward emodin, which agreed with the finding that emodin affected the cells through HAS2-HA-CD44/RHAMM pathway. Nevertheless, if HAS1 is affected by emodin was not explored in this study.

HA-CD44/RHAMM signal was thought to be associated with the cell cycle in tissue and cells [36, 37]. Flow cytometry with propidium iodide (PI) staining is commonly used in a cancer cell study [38]. Here we found that emodin significantly increased cells in the G1/G0 phase, and decreased cells in the S and G2/M phase. Cells in the G0/G1 phase were supposed to stop growing for other biological activities [39], while the S/G2/M phase is for cell proliferation [40]. This result indicated that emodin arrested cell proliferation. HAS2 knockdown showed similar effects on the cell cycle, causing an increase in cells in the G1/G0 phase, and a decrease in cells in the S and G2/M phase. The knockdown of HAS2 also blocked the effect of emodin, suggesting that emodin affected the cell cycle of A549 through HAS2. cell cycle was the downstream targets of the emodin-HAS2-HA-CD44/RHAMM pathway. The subsequent study showed that the effect of the pathway on the cell cycle was mediated by cyclin proteins. As cyclin A regulates the S phase [41] and cyclin B regulates the G2 phase[42], emodin decreased cyclin A and B which results in the decrease in cells at the S/G2 phase. Cyclin C was proved to suppressed cancer [43], it is also a downstream target of emodin found by this study. In terms of G1 phase regulators, we tested the cyclin D and cyclin E [44]. Our data revealed that both cyclin D and cyclin E raised after emodin exposure, which consistent with the increase in the G1 phase cells. These cyclin proteins were not changed by emodin exposure when HAS2 was knocked down suggesting that emodin affected the cell cycle through regulating HAS2. In addition, cancer stem cells might also play a role in the effect of emodin[45]. Considering the role of the HA in lung cancer, the potential effect of emodin might also involved cancer migration, some cell motility markers (FAK protein and MMPs) can be determined to explore the effect of emodin on cancer migration.

## Conclusions

In balance, this study demonstrated that emodin suppresses non-small lung cancer cells by cell cycle regulation. We suggested that the HAS2-HA-CD44/RHAMM pathway is one of the pharmacological mechanisms of emodin, but whether HAS2 is a direct or indirect target is still unclear, more work regarding the upstream target of emodin is required. Given the clinical value of emodin as a potential anti-cancer drug, our study is conducive to the development of emodin as a novel therapeutic option for lung cancer.

## Data Availability

All data generated or analyzed during this study are included in this published article.

## Abbreviations

NSCLC: Non-small cell lung cancers
HAS: Hyaluronan synthase
RHAMM: Hyaluronic acid-mediated motility
